# Using the Technology Acceptance Model to Identify Factors That Predict Likelihood to Adopt Tele-Neurorehabilitation

**DOI:** 10.3389/fneur.2020.580832

**Published:** 2020-12-02

**Authors:** Marlena Klaic, Mary P. Galea

**Affiliations:** ^1^Allied Health Department, Royal Melbourne Hospital, Parkville, VIC, Australia; ^2^Department of Medicine (Royal Melbourne Hospital), University of Melbourne, Parkville, VIC, Australia

**Keywords:** stroke, neurorehabilitation after stroke, tele-neurorehabilitation, technology—ICT, telehealth acceptance

## Abstract

Tele-neurorehabilitation has the potential to reduce accessibility barriers and enhance patient outcomes through a more seamless continuum of care. A growing number of studies have found that tele-neurorehabilitation produces equivalent results to usual care for a variety of outcomes including activities of daily living and health related quality of life. Despite the potential of tele-neurorehabilitation, this model of care has failed to achieve mainstream adoption. Little is known about feasibility and acceptability of tele-neurorehabilitation and most published studies do not use a validated model to guide and evaluate implementation. The technology acceptance model (TAM) was developed 20 years ago and is one of the most widely used theoretical frameworks for predicting an individual's likelihood to adopt and use new technology. The TAM3 further built on the original model by incorporating additional elements from human decision making such as computer anxiety. In this perspective, we utilize the TAM3 to systematically map the findings from existing published studies, in order to explore the determinants of adoption of tele-neurorehabilitation by both stroke survivors and prescribing clinicians. We present evidence suggesting that computer self-efficacy and computer anxiety are significant predictors of an individual's likelihood to use tele-neurorehabilitation. Understanding what factors support or hinder uptake of tele-neurorehabilitation can assist in translatability and sustainable adoption of this technology. If we are to shift tele-neurorehabilitation from the research domain to become a mainstream health sector activity, key stakeholders must address the barriers that have consistently hindered adoption.

## Stroke

Great advances have been made in acute stroke management, which has led to a marked decrease in mortality rates (1). However, incidence remains high with almost 14 million new strokes occurring annually and more than 80 million prevalent cases globally in 2016. The annual cost to society for first-ever stroke in Australia is AUD $5 billion and in the United States USD $50 billion and includes hospitalization, informal care and loss of productivity (2, 3).

Stroke is the main cause of acquired disability in the adult population with high numbers of survivors experiencing sensorimotor impairment, reduced cognition, and reduced function (4–7). There is strong evidence showing that neurorehabilitation in the acute, subacute, and chronic phases of recovery improves patient outcomes across numerous domains including activities of daily living and health-related quality of life (8–13). Improvement in function and subsequent reduction of disability, by as little as 1-point on the Modified Rankin Scale (mRS), can reduce the costs of care by 85% (2). Despite the evidence of the effectiveness of neurorehabilitation and the potential to reduce burden of care and associated costs, access to rehabilitation is inequitable. A recent audit of acute stroke care in Australia found that only 39% of patients admitted with a primary diagnosis of stroke were assessed for rehabilitation yet 75% were found to have rehabilitation needs (14). This suggests that a large number of Australian stroke survivors may be missing out on the opportunity to maximize their recovery. An Australian study exploring rehabilitation referral patterns for stroke survivors found there were significant variations in selection resulting in inequitable access to rehabilitation (15–17). The reasons for the variation in referrals for neurorehabilitation are multiple and include clinical and non-clinical factors such as reduced workforce capacity and limited access to rehabilitation beds requiring a prioritization approach (17).

Factors that impact on the provision of specialize neurorehabilitation are common across both developing and developed countries (14–18). In Australia, funding models typically emphasize reducing length of stay in an effort to reduce the cost of an episode of rehabilitation. In developing countries, access to organized stroke care, particularly neurorehabilitation, is limited (1, 19, 20). The need for alternative models of neurorehabilitation that are effective and efficient and can overcome current barriers has become an urgent priority, particularly in the more recent context of the COVID-19 pandemic where the demand for remote healthcare has increased rapidly. Telehealth strategies have shown great potential globally as an effective strategy to improve accessibility to healthcare (21, 22). Neurorehabilitation delivered using a telehealth platform, known as tele-neurorehabilitation, may overcome some of the barriers evident in more traditional, center-based models of care. This is particularly true for low and middle-income countries where access to specialized health and rehabilitation services is limited but information and communication technologies (ICT) are readily available and commonly used (23).

## Tele-neurorehabilitation

Tele-neurorehabilitation refers to a model of care that uses ICT to deliver clinical rehabilitation and education to patients with a neurological condition at a remote location, such as the patient's home (24, 25). There is a broad range of ICT that may be used in tele-neurorehabilitation from simple devices such as telephones and videoconferencing, up to more complex sensor-based systems with inertial measurement units (25–28).

The number of studies exploring effectiveness of tele-neurorehabilitation has grown over the last 10 years. This increased focus reflects advances in ICT and the growing need to find efficient, effective and economical models of care in the context of fiscally constrained healthcare settings. Laver et al. recently completed a systematic review of tele-neurorehabilitation for stroke services which included evidence from 22 randomized controlled trials with a total of 1,937 participants (29). The studies encompassed a large range of interventions such as mobility retraining, communication therapy and upper limb programs. The technologies used were equally varied and included telephone follow ups, electrical stimulation, IMU sensors and a virtual online library. The authors of the review found there was moderate-quality evidence that tele-neurorehabilitation for stroke survivors achieves results equivalent to usual care for activities of daily living, depressive symptoms, and health related quality of life. However, Laver et al. noted that the studies included in the systematic review did not address feasibility of ICT from the perspective of either the participants or the prescribing clinicians. This raises questions regarding what type of patient is most appropriate for a tele-neurorehabilitation program, how much training is needed for both user and prescriber and what infrastructure is necessary to support sustainable implementation of this model. The large body of research on implementation science suggests that a theoretical model can provide a framework to guide both implementation and evaluation of tele-neurorehabilitation and potentially enhance sustainable adoption of this model (30).

## Tele-neurorehabilitation and Technology Acceptance

The technology acceptance model (TAM) is one of the most widely utilized theoretical models explaining an individual's intention to use new technology (31, 32). The first iteration of the TAM was developed in the 1980's and proposed that a person's intention to use and subsequent use of technology can be predicted two beliefs: (1) their perception of how useful the technology is, and (2) their perception as to whether it is easy to use. A large body of research on the TAM found that it consistently predicted 40% of the variance in the intention to use and subsequent use of technology (31). Twenty years later, the TAM was extended to become the TAM2 and included output quality, results demonstrability, job relevance, subjective norm and perceived ease of use as determinants of perceived usefulness. Another model, the determinants of perceived ease of use, was developed at the same time and included factors that anchor beliefs about technology, including computer self-efficacy, computer anxiety, computer playfulness and perceptions of external control (31). Furthermore, experience and voluntary use of the technology were considered to be factors that moderated perceived usefulness.

Most recently, the TAM2 and the determinants of perceived ease of use have been combined to become the TAM3. This integrated model proposes that perceived usefulness is influenced by a number of factors including the quality of output from the technology and how relevant it is to the needs of the user. Perceived ease of use is determined by the person's beliefs about their own skills and includes computer self-efficacy and anxiety. Importantly, both perceived usefulness and perceived ease of use can be mediated through external factors such as increased practice / experience using the technology and adequate resources to support the person's use of technology (31).

There is a significant body of research on application of all iterations of the TAM in health settings, particularly in relation to the adoption of electronic health records and telehealth (33, 34). A recent study used the TAM to predict if a group (*n* = 325) of Canadians would use electronic medical health records to manage health information such as making future appointments (35). The authors found that perceived ease of use was the strongest predictor of perceived usefulness. The users' prior experiences with technology, needs and values all correlated with intention to use the electronic medical health record. Another study exploring patient uptake of electronic health records found that difficulties logging in and a complex user interface impacted on adoption (36). Despite the growing evidence-base using the TAM to predict user adoption of technology, none of the published studies focus on tele-neurorehabilitation. The aim of this perspective is to explore if published studies on tele-neurorehabilitation can be mapped onto the variables in the TAM3.

## Methods

A systematic mapping review approach was selected as the intention was to describe and categorize the body of tele-neurorehabilitation evidence using the TAM3 framework. A traditional systematic review aims to identify and assess the quality of published literature in order to answer a very specific question. By contrast, a systematic mapping review characterizes the literature and catalogs it according to a criteria or framework or model. In this study, the published literature on tele-neurorehabilitation will be described and categorized using the TAM3 framework. A systematic mapping review process is particularly useful when the topic area is broad and the quality and range of studies is diverse (37–39). This approach can provide information about knowledge gaps and therefore direct future research, including systematic reviews.

The methods applied to a systematic mapping review process are as follows: (1) literature search, (2) literature selection, and (3) literature mapping to the TAM3.

### Literature Search

Databases relevant to the health sciences were searched including CINAHL (EBSCO), PsycINFO (EBSCO), PubMed (National Center for Biotechnology Information), and SCOPUS. Search terms were telerehabilitation or tele-rehabilitation or telehealth or remote rehabilitation AND stroke or cerebrovascular accident or CVA AND home or remote. Limitations included English-language, adult population and peer-reviewed papers. The search date was for studies published from 2000 to July 2020.

### Literature Selection

The aim of this study was to determine if the existing literature on tele-neurorehabilitation could be mapped using the TAM3, with a specific focus on the user experience. Therefore, studies which included any information on patient or therapist experience of tele-neurorehabilitation, were a particular target. Following removal of protocols, center-based interventions and systematic reviews, a total of 22 studies were identified. Table 1 presents data extracted from the studies including aims, population and outcomes. The studies included pre/post-studies, evaluation of devices, and qualitative exploration of user experience with tele-neurorehabilitation. Participants were stroke survivors in acute, sub-acute or chronic phases of recovery and varied in their impairments. Consequently, the tele-neurorehabilitation interventions included sit-to-stand practice, communication therapies, psychosocial interventions and activities of daily living practice. The type of ICT used also varied widely from telephones to apps with associated sensor data.

**Table 1:** Data extraction for studies included in the mapping review.

**Author (date, country)**	**Study design sample**	**Study aim**	**Intervention**	**Outcomes measured**	**Results**
Burdea et al. (40)	Pre/post Stroke survivors and their caregivers (*n* = 8 + 8)	To evaluate the feasibility of a tele-neurorehabilitation system developed for the study	4-weeks (20 sessions) participating in serious gaming with Grasp game controller	Motor function and impairment Emotion and cognition Survey of user experience	High rate of compliance Improvement in mood and cognition Participants had an overall positive attitude to the system Both carers and participants scored technical problems as the lowest
Chen et al. (41)	Qualitative Stroke survivors *N* = 13 participants	To investigate patient perceived benefits of and barriers to using a telerehabilitation system at home	6-weeks using a home-based telerehabilitation system with serious gaming 18 sessions supervised 18 sessions unsupervised	Semi-structured interviews exploring attitudes, motivation and usage	Perceived improvement in physical abilities, psycho-social health and well-being Participants intended to continue to use the system provided improvements in games and progress feedback were made
Cherry et al. (42)	Qualitative Stroke survivors *N* = 10	To determine participants' general impressions about the benefits and barriers of using robotic therapy devices for in-home rehabilitation	2-h daily robotic assisted therapy for a maximum period of 3-months	Direct observation In-depth semi structured interviews exploring the user experience	Benefits included increased mobility, sense of control over therapy and outlet for stress and tension Barriers were donning the hardware (arm device) and technical difficulties
Cronce et al. (43)	Case Report Stroke survivor *N* = 1	To evaluate the feasibility of a virtual rehabilitation system developed for the study	7 ×30-min training sessions using the VR system and serious gaming	Questionnaire exploring system use	Easy to use system that was highly engaging and motivating
Deng et al. (44)	Pilot RCT Stroke survivors Experimental *N* = 8 Control *N* = 8	To explore feasibility of using telerehabilitation to improve ankle dorsiflexion and to compare complex vs. simple movements of the ankle	4-weeks of telerehabilitation using a computerized system	Gait 10-meter walk test fMRI Participant feedback	Improved ankle dorsiflexion Difficulties donning hardware but overall
Deutsch et al. (45)	Case Report Stroke Survivor Clinician (*N* = 1 + 1)	To describe the outcomes of using motor imagery via a telerehabilitation platform	3 ×45–60-min sessions over 4-weeks using motor imagery delivered in the home with telerehabilitation	Imagery ability Motor behavior Fugl-Meyer Timed up and Go Questionnaire on system usability	Improvement in gait and balance Both patient and clinician found the system useful Lowest score for functions and capabilities of the system
Dodakian et al. (46)	Pre/Post Stroke survivors *N* = 12	To assess feasibility and motor gains of a telerehabilitation system developed for the study	28-days of home-based telerehabilitation delivered in 2 ×14-day blocks System consisted of specialized computer, table and set up for serious gaming	Vital signs Arm motor function Mood QoL Survey of patient experience with the technology	Improvement in arm motor function compliance and satisfaction with the system Improved stroke prevention knowledge No correlation between computer literacy and outcomes
Ellington et al. (47)	Pre/Post Stroke survivors *N* = 14	To investigate the behavioral intention to use a virtual system for practicing instrumental activities of daily living	4 ×1 h sessions using affected upper limb to practice two virtual activities e.g., meal preparation	Questionnaire based on the TAM Semi-structured interview	Positive attitude and intention to use technology Relationship between perceived usefulness and intention to use
Flynn et al. (48)	Case report Stroke survivor *N* = 1	To explore the use of a low-cost virtual reality device	20 ×1-h sessions using a low-cost virtual reality device with associated serious gaming	Fugl-Meyer Timed up and go Daily logs of system use In-depth interview	Improvement in motor function, mood, mobility and gait Reported the system was motivating
Kurland et al. (49)	Pre/Post Stroke survivors *N* = 21	To determine if a table-based home practice program could enable maintenance of treatment gains in post-stroke aphasia	6-month home practice program with weekly teletherapy sessions	% accuracy on naming Boston naming test	Greater number of training sessions with the technology resulted in fewer gains in naming accuracy
Lai et al. (50)	Pre/Post Stroke survivors *N* = 21	To evaluate the feasibility of using videoconferencing for community-based stroke rehabilitation	8-week intervention delivered at a community center for seniors via videoconferencing. Included education modules, exercise and psychosocial support	Balance Self esteem Stroke Knowledge Mood ADL Focus group discussions exploring satisfaction	Improvements in balance, self-esteem, stroke knowledge and quality of life. 67% rated clinical effectiveness of the system as good
Langan et al. (51)	Cross-sectional study Therapists *N* = 107	To examine the extent to which physical and occupational therapists use technology in clinical stroke rehabilitation programs	N/A	Survey measuring use of technology	Poor use of technology even when available
Piron et al. (52)	Pilot study Stroke survivors *N* = 10	To compare degree of satisfaction of patients using virtual reality therapy programmed at home with those using the same system in a hospital setting	1-h of rehabilitation daily for 1 month involving virtual tasks practiced in a VR system	Fugl-Meyer scale Questionnaire measuring degree of satisfaction	High compliance Tele-neurorehabilitation group had a lower score for therapist explanation of the treatment, higher outcome for UL motor
Rogerson et al. (53)	Mixed-methods evaluation Chronic stroke survivors *N* = 19	To assess the feasibility and acceptability of a smart home system that monitors users' activity	Installation of a system and participant education on how to use it	Interview on user experience of the system	The technology gave peace of mind Engagement with the system was variable
Seo et al. (54)	Pre/Post Chronic stroke survivors *N* = 10	To assess usability of a virtual reality rehabilitation system	Not described	Survey of user experience	Preference for easy to use games
Simpson et al. (55)	Pre/Post Stroke survivors *N* = 8	To investigate the feasibility of a phone-monitored home exercise program for the upper limb following stroke	8-week home exercise program with weekly telephone contact with therapist	Chedoke arm and hand inventory Motor activity log Grip strength Occupational performance Feasibility outcomes	Did not achieve exercise adherence or goal rates Motor improvement maintained at 3 and 6 month follow up
Simpson et al. (56)	Pre/Post Stroke survivors *N* = 10	To determine whether telerehabilitation is feasible in monitoring adherence and progressing functional exercises at home	4-weeks of telerehabilitation using an app with serious gaming and sensor system to monitor movements	Short physical performance battery (SPPB) Timed sit-to-stand test Satisfaction questionnaire	High compliance with the program High ratings for system usability, enjoyment and perceived benefits Improvement in SPPB
Standen et al. (57)	Prospective cohort study Stroke survivors *N* = 17	To investigate patient use of a low-cost virtual reality system	Equipment left in patient homes for 8-weeks with advice to use 3 times per day for maximum 20 min	Duration, frequency and intensity of use	Lack of familiarity with technology impacted use
Threapleton et al. (58)	Cross-sectional study Acute stroke survivors (*N* = 4) Chronic stroke survivors (*N* = 8) Occupational therapists (*N* = 13)	To explore the value of virtual reality in preparing patients for discharge following stroke	Demonstration of a virtual home application prior to the interview	Semi structured interviews	Occupational therapists felt the system had the potential to educate and engage the patients in preparing for discharge home but may not be suitable for all patients Stroke survivors felt the system was not representative of their own homes
Triandafilou et al. (59)	Pre/Post Stroke survivors *N* = 15	To evaluate a virtual environment system developed for the trial and compare to an existing virtual reality system and a home exercise program (HEP)	1-week participation in each of the three interventions (total of 3-weeks)	Arm displacement Survey to measure participation and satisfaction	Low satisfaction with time spent in training for the VR system Preference for HEP over the other two systems
Warland et al. (60)	Pre/Post Chronic stroke *N* = 12	To establish feasibility, acceptability and preliminary efficacy of an adapted version of a commercially available, virtual-reality gaming system for upper-limb rehabilitation	9 ×40-min exercise sessions utilizing the system for 30 days per week over 3-weeks	Semi structured interview to explore feasibility and acceptability Fugl-Meyer Assessment Action research arm test Motor activity log Participation	High level of enjoyment Improvement in all motor and function outcomes
Woolf et al. (61)	Quasi-randomized controlled feasibility study Chronic stroke survivors with aphasia *N* = 21	To test the feasibility of a randomized controlled trial comparing face to face and remotely delivered word finding therapy for people with aphasia	8 ×1 h therapy delivered using videoconferencing technology compared to face to face therapy and an attention control condition	Word retrieval Recruitment and attrition rates Participant observation and interviews Treatment fidelity	Treatment fidelity was high Compliance and satisfaction with the intervention were good Picture naming improved but not naming in conversation

### Literature Mapping to TAM3

**Figure 1:**
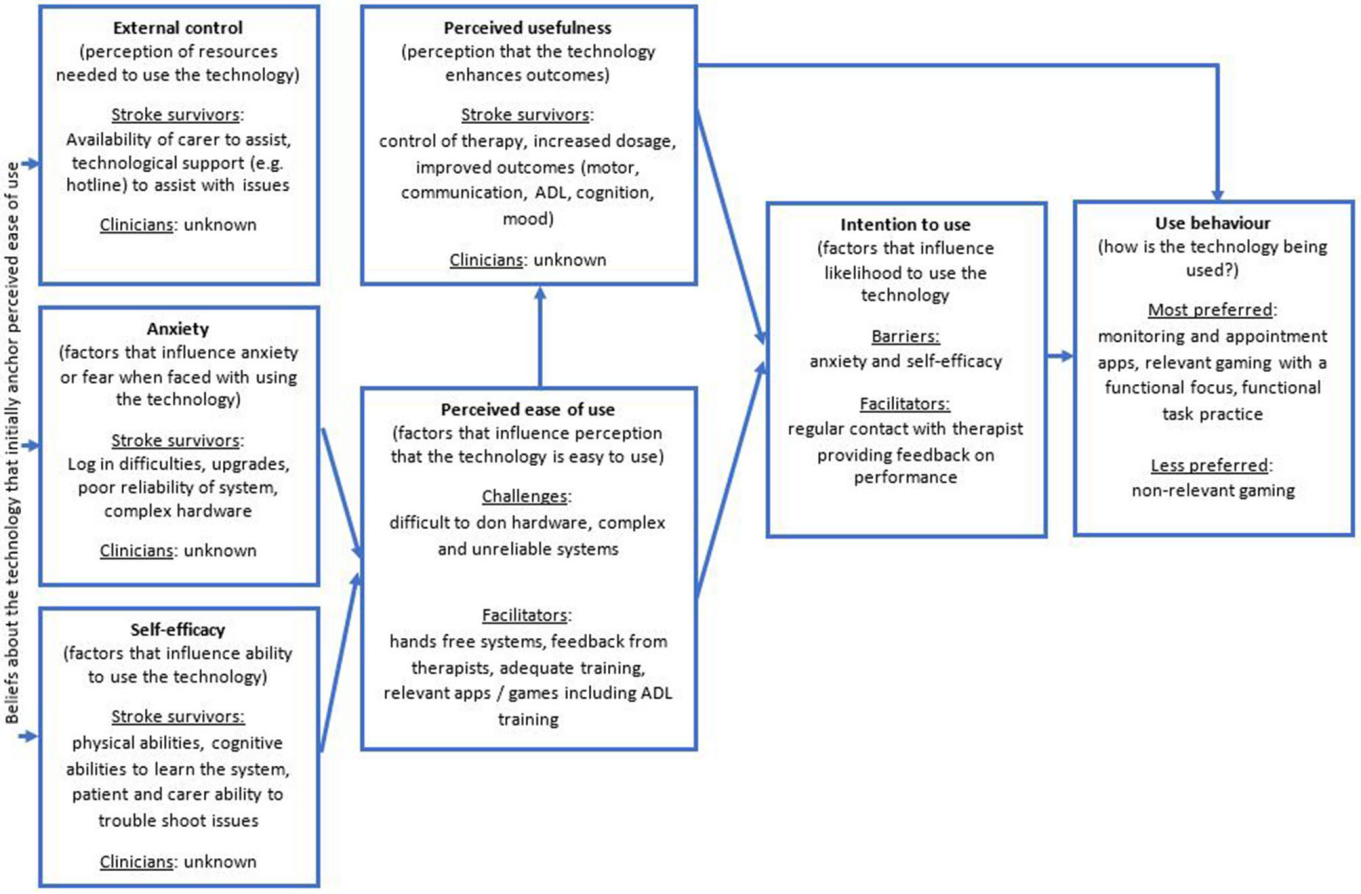
Result of mapping the 22 studies to the variables of the TAM3 (31).

All 22 studies were readily able to be mapped on to the TAM3 and revealed patterns in relation to the barriers and facilitators for tele-neurorehabilitation. Figure 1 displays the findings using the TAM3 model which is expanded on in the following sections.

## Results

### Tele-Neurorehabilitation and Perceived Usefulness

#### Do Stroke Survivors and Clinicians Perceive That Tele-Neurorehabilitation Will Be Beneficial?

The majority of studies found that tele-neurorehabilitation interventions were not inferior to conventional center-based models of care (41, 42, 46, 47, 50, 54–56, 60). Patients and carers/family reported subjective improvements in communication, gait, activities of daily living, and motivation. Only one study found that patients preferred a conventional home exercise program over tele-neurorehabilitation due to the perception that the tele-neurorehabilitation was too complex (59). None of the studies explored therapist perceptions of the usefulness of tele-neurorehabilitation, particularly in comparison to conventional models of center-based neurorehabilitation.

### Tele-Neurorehabilitation and Perceived Ease of Use

#### Do Stroke Survivors and Clinicians Perceive That Tele-Neurorehabilitation Is Easy to Use?

A number of studies found that participants enjoyed gaming technology associated with some of the tele-neurorehabilitation interventions and found them easy to use, engaging and motivating (41, 43, 47, 48, 52, 54). Experiences with both hardware and software had a marked effect on the user perception of tele-neurorehabilitation. For example, hands-free systems were perceived as easier to use (60) than those that required the participant to don/doff splints, sensors, and other similar hardware (40–42, 45, 56, 57).

Low self-efficacy related to the tele-neurorehabilitation system used was a frequently reported problem that affected perception of ease of use, compliance, and subsequent intention to continue using the system in the future (57, 60). Conversely, prior experience with relevant technology, such as computers, correlated to improved compliance and perceptions of ease of use (46, 53).

Anxiety and frustration with tele-neurorehabilitation was apparent when more complex ICT and hardware was used (42, 44, 46). Unreliable internet bandwidth, and technical issues which were not easily resolved further contributed to anxiety and perceptions of ease of use (46). Studies where the ICT was familiar (e.g., telephones) and consisted of easy to understand tasks (e.g., sit-to-stand practice) appeared to reduce anxiety secondary to the perception that they were easier to use (47, 56).

Increased exposure and practice with tele-neurorehabilitation systems improved compliance and reduced anxiety related to low confidence and proficiency with technology (46, 48, 49, 59). However, the amount of experience necessary to reduce technology-related anxiety remains unclear with studies reporting variable amounts of time spent on training participants and therapists. Conversely, one study found there was a correlation between number of training sessions to achieve proficiency in using the technology and poorer outcomes, indicating that the technology may not be suitable for all disorders or patients (49).

The most commonly reported external variable necessary to support engagement in tele-neurorehabilitation was the presence of a carer/family member (44, 46, 61, 62). This was the case irrespective of the nature of intervention being delivered or the type of ICT being used.

### Tele-Neurorehabilitation and Behavioral Intentions

#### Are Stroke Survivors and Clinicians Motivated or Willing to Exert the Effort to Engage in Tele-Neurorehabilitation?

A number of studies found that participants reported an intention to continuing engaging in tele-neurorehabilitation, with some provisos, including a request for easier and more reliable technology and access to their performance results (40, 41, 47, 52, 53).

### Tele-Neurorehabilitation and Use Behavior

#### How Are Stroke Survivors and Clinicians Using Tele-Neurorehabilitation?

Tele-neurorehabilitation with a focus on relevant, easy to use components was rated more highly by participants than complex systems with multiple componentry (46, 53). For example, appointment reminder systems, monitoring apps and ADL focused gaming was selected more often than motor-based gaming. Participants also preferred tele-neurorehabilitation systems where the therapist could observe and provide feedback and encouragement via a videoconferencing or other interactive system (41, 45, 54, 56).

## Discussion

It is anticipated that the demand for neurorehabilitation will continue to grow due to an aging population and high incidence rates of diseases such as stroke. Rehabilitation resources in both developing and developed countries are limited and the need to find alternative yet effective and efficient models is imperative. Tele-neurorehabilitation has great potential to increase accessibility to rehabilitation for individuals with neurological impairments. However, consideration must be given for both human and ICT factors that can hinder or facilitate adoption of tele-neurorehabilitation.

A review of the published evidence on tele-neurorehabilitation through the lens of TAM3 reveals that perception of ease of use is influenced by the user's belief that they have the requisite skills and ability to use the technology (computer self-efficacy) and the degree of apprehension or fear they experience when faced with learning to use the technology (computer anxiety). Easy to use ICT and adequate experience using the technology assists the user to adjust their beliefs about computer self-efficacy and reduces computer anxiety (46, 63). Some studies found that patients were open to and excited about tele-neurorehabilitation but experienced numerous technological malfunctions which increased computer anxiety and reduced perceived enjoyment (64). Nearly all studies found that carers were critical to ensure that patients were able to overcome barriers related to system set-up, thus reducing computer anxiety (63).

There is little to no evidence on the feasibility of tele-neurorehabilitation for the “typical” stroke survivor who is likely to have cognitive impairment. Published studies have found that patients with cognitive impairment can benefit from computer training programs, suggesting that at least some of these patients may have computer self-efficacy and be appropriate for a tele-neurorehabilitation intervention. There is little to no evidence on how much experience or practice and training with a system is needed for the stroke survivor to become a confident user. Understanding the type and frequency of training necessary to establish and maintain computer self-efficacy would contribute to a more informed implementation and sustainable adoption of tele-neurorehabilitation.

Although gains have been made in design of ICT to potentially enhance tele-neurorehabilitation, barriers to adoption that were identified more than 20 years ago remain apparent today (65). These barriers are relevant for both patients and prescribing clinicians and include poor computer self-efficacy, high computer anxiety, low perception of usefulness and a belief that the technology is not user-friendly (65, 66). If we are to realize the full potential of tele-neurorehabilitation, it is of critical importance that we approach the topic using a validated and well-tested theoretical framework to guide and evaluate implementation. This would make a significant contribution to the evidence-base on tele-neurorehabilitation.

## Data Availability Statement

The original contributions presented in the study are included in the article/supplementary material, further inquiries can be directed to the corresponding author/s.

## Author Contributions

MK and MG conceived the ideas presented. MK completed the initial draft of the manuscript which was reviewed and edited by MG resulting in the final manuscript. All authors contributed to the article and approved the submitted version.

## Conflict of Interest

The authors declare that the research was conducted in the absence of any commercial or financial relationships that could be construed as a potential conflict of interest.
